# Responses of the native species *Sparganium angustifolium* and the invasive species *Egeria densa* to warming and interspecific competition

**DOI:** 10.1371/journal.pone.0199478

**Published:** 2018-06-20

**Authors:** Hongwei Yu, Nan Shen, Siqi Yu, Dan Yu, Chunhua Liu

**Affiliations:** The National Field Station of Freshwater Ecosystem of Liangzi Lake, Department of Ecology, College of Life Sciences, Wuhan University, Wuhan, P.R. China; Shandong University, CHINA

## Abstract

Climate change, especially warming temperatures, may increase invasion and modify the ecological impacts of invasive species by enhancing their ability to compete. To test the effects of warming on invasive plants, a mesocosm experiment was conducted to study competition between the invasive plant *Egeria densa* and the native hygrophyte *Sparganium angustifolium* under simulated warming conditions in a greenhouse. These two species were grown in monoculture (no competitor control) or mixed culture (competitor control) for two months under different temperature conditions (warming treatment or no-warming treatment). In *S*. *angustifolium*, the higher temperatures led to a shorter root length and significantly increased the aboveground traits of ramets, the total biomass, and the RGR (relative growth rate) but had no effect on the aboveground traits of genets. Growth in mixed culture significantly decreased the *S*. *angustifolium* ramet height under warmer conditions and significantly reduced the ramet root length, ramet number, genet biomass, root-to-shoot ratio and RGR of *S*. *angustifolium* under natural temperature conditions. All the morphological, biomass and growth traits of *E*. *densa* except for the root-to-shoot ratio were significantly increased by the warmer temperatures and decreased by growth in mixed culture. The RCI and RII of *E*. *densa* in both the no-warming and warmer environments were two and three times greater than those of *S*. *angustifolium*, whereas the ACI values for the two species were similar. Thus, *S*. *angustifolium* was a better competitor than *E*. *densa* under both temperature conditions. These results suggest that although the superior competitive ability of native species can inhibit *E*. *densa* growth, the performance of this species will be enhanced under future climate warming in cold regions.

## Introduction

Climate change and biological invasion are two of the most pervasive aspects of global environmental change[1]. The global mean surface air temperature has increased by 0.74°C over the last half century and is predicted to increase by a further 1.4–5.8°C over the period from 1990 to 2100 as a result of climate change[2]. Temperature is one of the critical factors influencing plant growth and distributions[3], and elevated temperature significantly affects plant physiological functions and morphological characteristics[4]. Global warming is likely to promote the expansion of many invasive species[5, 6] and thus influence regional biodiversity, phenological plasticity, genetic composition, interspecific relationships and ecosystem processes[7, 8]. With the wide variety of global changes currently occurring, it is important to better understand the relationship between biological invasions and environmental change[7, 9, 10].

Temperature increases related to global warming may have drastic effects on aquatic plants[11] because the ambient temperature (of the water and sediment) influences the growth and morphology of aquatic plants by affecting their physiology, including the germination of seeds, the initiation and rate of seasonal growth, and the onset of dormancy[12, 13]. As a result of global warming, invasive aquatic plants have gradually become one of the major threats to freshwater biodiversity[14] and have already reduced native species richness and disturbed the balance of the ecosystem[15, 16].

High competitive ability in a species is regarded to be an invasive trait[17]. Most studies have reported that invasive species are superior competitors over native species[18, 19]. Consequently, invading species are more successful when the levels of competition between the invader and neighboring plants in the recipient community are low[20]. High morphological plasticity and physiological mechanisms may elevate the competitive ability of invasive aquatic plants[21–25]. Invasive species are frequently superior competitors that may cause the extinction of native species or replace and exclude them from habitats[12]. For example, *Potamogeton crispus*, *Elodea canadensis* and *Myriophyllum spicatum* exhibit higher photosynthetic efficiency and nutrient uptake than native species and then rapidly propagate at the water surface, reducing the light and space for growth available to native plants [22, 24]. In addition, plant functional traits may increase invader abundance (invasiveness) and their impacts in changing environments[26], and invasive plant species show functional traits that are consistent with high resource acquisition[27].

However, environmental factors may influence the intensity of competition. For example, nutrients can determine the relative competition among plant species[28]. Experimental warming changes the competitive response and the effect of competition on plant communities [29], which suggests that climate warming may affect the local community structure and the intensity of competition between native and invasive plants[6, 30, 31], although Verlinden et al. [30, 32] found that warming did not modify the competitive balance between three highly invasive plants and their native competitors. The responses of the native-invasive interaction to warming cannot always be extrapolated from the responses of plants grown in monoculture or those of several invasive species; more experiments that take into account species interactions in general should be conducted[30, 32]. In addition, few studies have investigated competition along with the effects of temperature warming in invasive and native macrophytes, and more such studies may help us to understand biological invasions by aquatic plants.

Therefore, we designed an experiment to simulate the influence of warming on the growth of the invasive aquatic plant *Egeria densa* (which has survived in one small reservoir in a cold temperate zone in China over the last few years) and the native aquatic plant *Sparganium angustifolium* (which is the dominant aquatic plant in the cold temperate zone). Global warming could increase the spread of invasive species to higher latitudes and intensify the effects of competition on native species[6]. In terms of global warming, the increasing ambient temperature is a crucial component[7]. Thus, we used mixed cultures to evaluate how the growth of these two species and the competitive relationships between them species are affected by warming. We aimed to address the following hypotheses: (1) Warming will promote the growth of *E*. *densa* and *S*. *angustifolium*. (2) Competition with native species will inhibit the growth of *E*. *densa*.

## Materials and methods

### Plant materials

#### *Sparganium angustifolium* Michx

*Sparganium angustifolium* (Sparganiaceae) is a perennial stoloniferous herbaceous plant native to Heilongjiang, Xinjiang, Inner Mongolia, China[33]. This species has two growth forms, submerged and emergent, which occur in habitats with different water levels. It can spread horizontally above the sediment surface, and it usually allocates more resources aboveground, developing slender floating leaves to form a canopy on the water surface in northern China.

#### *Egeria densa* Planch

*Egeria densa* (Hydrocharitaceae) is a submerged freshwater perennial herbaceous plant found in both lentic and lotic environments that is native to South America[34, 35]. It was introduced and has become weedy in North America, Australia, Asia and Europe and countries in other locations[35]. *E*. *densa* relies mainly on vegetative propagation through stem fragments[36]. The invasion by *E*. *densa* has affected the hydrophyte community structure and obstructed water channels and hydroelectric turbines[35, 37]. Several large-scale surveys of macrophyte floras have indicated that *E*. *densa* can repress or displace native hydrophytes in different invaded areas[34, 38]. In recent years, this species was introduced to China for ornamental purposes. It is common in aquarium markets and landscape water bodies and has escaped into natural habitats in China. *E*. *densa* can temporarily survive under ice and can overwinter to regenerate by using stored starch in its leaves and old stems during the autumn [36, 39, 40]. Therefore, *E*. *densa* may further invade the cold temperate zone of China and may affect the growth performance of native aquatic plants. Although some experiments have assessed the competitive relationship between *E*. *densa* and native hydrophytes[37, 38], they were usually conducted in already invaded tropical or subtropical regions rather than in cold temperate regions that may potentially be invaded by this species. With the intensification of global warming, we should predict the potential invasion by invasive aquatic plants in freshwater ecosystems at high latitudes and in cold regions.

### Experimental design

This study was conducted in Arongqi County, Inner Mongolia, China (48°10.883′ N, 123°22.699′ E; altitude: 206 m). The Alun River of Arongqi County is a perennial flowing water body, and *S*. *angustifolium* occurs in its submerged form in this river. We collected individuals of submerged *S*. *angustifolium* from the Alun River and individuals of *E*. *densa* from a natural pond in Dalian, China (39°49.336′ N, 122°04.866′ E). The use of natural sediments rather than culture solutions as the source of nitrogen, phosphorus and micronutrients largely prevents the occurrence of algal blooms[39]. Thus, Alun River sediment and water were used in our experiment. All of the collected plant materials were precultivated in plastic buckets with 15 cm of Alun River sediment and 40 cm of Alun River water (soil: mean ± SE, 0.29 ± 0.03 mg.g^-1^ N, 0.53 ± 0.02 mg.g^-1^ P, 31.64 ± 1.12 mg.g^-1^ organic material content; water: 0.86 ± 0.14 mg.L^-1^ N, 0.16 ± 0.04 mg.L^-1^ P) for approximately 75 days in the natural environment. On July 15, 2016, 48 clonal seedlings of *S*. *angustifolium* (height: approximately 20 cm; initial dry mass: mean ± SE, 0.134 ± 0.011 g) and 48 clonal ramets of *E*. *densa* (height: approximately 12 cm; initial dry mass: mean ± SE, 0.077 ± 0.008 g) were selected for the experiment.

The global mean surface air temperature is predicted to increase by a further 1.4–5.8°C over the period from 1990 to 2100[2]. The mean water and air temperature in the greenhouse was 2.6°C and 4.6°C higher, respectively, than that in the natural environment. To avoid the influence of intraspecific competition, the plant density treatment was conducted using a simple pair-wise experimental design that provides useful information about the effects of a treatment gradient on the outcome of competition[41]. We used additional monoculture treatments (no competitor controls) to assess the roles of various factors (e.g., initial plant size, herbivory) on the response of the target plant to interspecific competition and to identify the proportion of species affected by competition[42].

A randomized block design was used to test the effects of interspecific competition and the warming treatment on the growth performance of *E*. *densa* and *S*. *angustifolium*. The plants were either subjected or not subjected to interspecific competition using the following treatments: 1. Monoculture (no competitor control): *S*. *angustifolium* (each container had one plant); 2. Monoculture (no competitor control): *E*. *densa* (each container had one plant); 3. Mixed culture (competitor control): *S*. *angustifolium* × *E*. *densa* (each container had one plant per species) in the greenhouse and the same experimental setup for the control in the natural environment (no warming treatment: outside of the greenhouse). All plants were randomly assigned to each treatment. Each treatment was replicated 8 times. Forty-eight plastic buckets (diameter: 48 cm, height: 55 cm) were used as the experimental containers. Ten centimeters of Alun River clay (mean ± SE, 0.3 ± 0.02 mg.g^-1^ N, 0.56 ± 0.04 mg.g^-1^ P, 34.27 ± 1.95 mg.g^-1^ organic material content) was added to the bottom of each container, and then the containers were filled with Alun River water (0.77 ± 0.13 mg.L^-1^ N, 0.16 ± 0.02 mg.L^-1^ P). All the containers were randomly rearranged every 15 days to avoid the possible effects of environmental heterogeneity (such as light). The outside containers were covered with plastic cloths when it rained.

The experiment was conducted for two months, and the plants were harvested on September 16, 2016. Diurnal variation in temperature (air, water) and illumination were recorded using a temperature probe (L93-4, Loggertech, Co., Ltd., Hangzhou, CN) every half hour and with a digital lux meter (ZDS-10 W-2D, Jiadingxuelian, Co., Ltd., Shanghai, CN) every day, respectively, while the physical and chemical characteristics of the water were measured using a Professional Plus multiparameter instrument (YSI Incorporated, Ohio, USA), portable turbidimeter (2100Q, HACH, USA), colorimeter (DR890, HACH, USA), turbidimeter (2100P, HACH, USA), and digester (DRB200, HACH, USA) every week, and the mean daily temperature was calculated (Table 1) for each treatment combination during the experimental period. With the exception of the water and ambient temperatures, none of the physical or chemical properties significantly differed between the two environments (Table 1).

**Table 1:** Physical and chemical factor of water and microclimate parameters of the experiment during the experimental period, the boldface of a and b showed the results of variance analysis.

	Warming	No-warming
**Water temperature (°C)**	22.77±5.18^**a**^	20.17±6.19 ^**b**^
**Ambient temperature (°C)**	23.64±8.22 ^**a**^	19.58±7.39 ^**b**^
**Dissolved oxygen(mg/L)**	7.91 ± 0.21 ^**a**^	9.48 ± 1.04 ^**a**^
**Salinity**	0.1 ± 0.003 ^**a**^	0.09 ± 0.003 ^**a**^
**pH**	8.56 ± 0.07 ^**a**^	8.84 ± 0.21 ^**a**^
**Total phosphorus(mg/L)**	0.14 ± 0.03 ^**a**^	0.15 ± 0.03 ^**a**^
**Total nitrogen(mg/L)**	0.79 ± 0.09 ^**a**^	0.86 ± 0.09 ^**a**^
**NH**_**3**_**-N(mg/L)**	0.05 ± 0.03 ^**a**^	0.06 ± 0.02 ^**a**^
**Photosynthetically active radiation(μmol.m**^**-2**^**.s**^**-1**^**)**	1082.43±67.91^**a**^	1083.5±68.39^**a**^

The two species have different morphological characteristics, for example, *S*. *angustifolium* has obvious genets and ramets, and no stem structures were formed in this experiment. It was difficult to distinguish the genets and ramets in *E*. *densa*, but this species has a complete structure (leaves, stems, and roots). Hence, during the harvest, *S*. *angustifolium* was divided into genets (mother plant: initial plant) and ramets (all new clonal ramets). The height and root length of the genets and ramets were measured. The stolon length and ramet number were measured and recorded. The plant height, root length and ramet number of *E*. *densa* were recorded, and then the tissues were separated into leaves, stems and roots. Each plant was dried at 70°C for 72 h and then weighed.

The formulas for data calculation were as follows:

1. Absolute competition intensity: ACI = Pmono–Pmix
2. Relative competition intensity: RCI = (Pmono − Pmix) / Pmono
3. Relative interaction index: RII = (Pmono − Pmix) / (Pmono + Pmix)
4. Relative growth rate: RGR (g.g^−1^.day^−1^) = [ln (total biomass)–ln (initial biomass)] / days
5. R/S ratio (g.g^−1^) = root mass / (leaf mass + stolon mass)

Pmono is the total biomass in the absence of competition (i.e., one-plant density treatment), and Pmix is the average biomass of a plant in each container in the presence of competition (i.e., multi-density treatments). RII has defined limits [-1, +1], is symmetrical around zero and is positive for competition and negative for facilitation[43]. ACI is an important indicator used for monocultures, and RCI is the direct ratio of monoculture to mixture performance[44]. RII is used to measure the relative intensity of interactions among multiple plant species[43–46].

### Data analysis

Analysis of covariance revealed no significant effect of the initial plant dry weight of *E*. *densa* (*F* = 0.046; *P* = 0.832) or *S*. *angustifolium* (*F* = 0.195; *P* = 0.663) on the total biomass. All the data met the assumptions of normality and homoscedasticity. One-way ANOVA with Tukey’s test for post hoc comparisons were used to check the physical and chemical differences between the elevated temperature treatment and the control. The total biomass and leaf and stem biomass of *E*. *densa* and the ACI were transformed using the function log_10_(x). We used two-way ANOVA to test the effects of temperature (warming or no warming) and interspecific competition (mono- or mixed culture) on the growth and morphology of each species. The ACI, RCI and RII were also analyzed by means of two-way ANOVA (temperature × species). Tukey’s test was used to examine the differences in trait values among the treatments. All data were analyzed using SPSS 22.0 (SPSS, Chicago, Illinois, USA).

## Results

### The effects of warming and competition on the growth of *E*. *densa* and *S*. *angustifolium*

Temperature had significant effects on the ramet height, genet and ramet root length, ramet number, total biomass, ramet biomass, and RGR of *S*. *angustifolium* (Table 2). Competition had significant effects on the ramet root length, ramet number, genet biomass and RGR of *S*. *angustifolium* (Table 2). The interactive effects of temperature and competition on ramet root length and the R/S ratio were significant (Table 2).

**Table 2:** Two-way ANOVA results for effects of temperature and competition on measures oyf growth and morphology of *S*. *angustifolium* and *E*.*densa*.

			Temperature (T)		Competition(C)		T x C	
			*F*_*1*,*28*_	*P*	*F*_*1*,*28*_	*P*	*F*_*1*,*28*_	*P*
***S*. *angustifolium***		**Genet height (cm)**	0.314	0.579	0.326	0.573	0.413	0.526
		**Ramet height (cm)**	55.196	**<0.001**	2.644	0.115	2.937	0.098
		**Genet root length (cm)**	15.019	**0.001**	0.229	0.636	0.627	0.435
		**Ramet root length (cm)**	9.683	**0.004**	27.269	**<0.001**	8.809	**0.006**
		**Stolon length (cm)**	0.565	0.459	0.213	0.648	1.816	0.189
		**Ramet number**	24.518	**<0.001**	5.364	**0.028**	0.638	0.431
		**Total biomass (g)**	76.916	**<0.001**	8.514	0.07	0.000	0.998
		**Genet biomass (g)**	0.496	0.487	10.546	**0.003**	0.055	0.816
		**Ramet biomass (g)**	117.17	**<0.001**	5.808	0.23	0.009	0.924
		**R/S ratio (g. g**^**-1**^**)**	0.221	0.642	3.872	0.059	16.58	**<0.001**
		**RGR (g. g**^**-1**^**. day**^**-1**^**)**	89.872	**<0.001**	11.211	**0.002**	0.986	0.329
***E*. *densa***		**Ramet number**	46.850	**<0.001**	11.712	**0.002**	0.648	0.427
		**Plant height (cm)**	45.945	**<0.001**	38.715	**<0.001**	6.342	**0.018**
		**Root length (cm)**	40.445	**<0.001**	6.175	**0.019**	0.652	0.426
		**Total biomass (g)**	27.012	**<0.001**	42.377	**<0.001**	0.009	0.926
		**Leaf biomass (g)**	26.280	**<0.001**	33.379	**<0.001**	0.011	0.916
		**Stem biomass (g)**	23.954	**<0.001**	53.733	**<0.001**	0.379	0.534
		**Root biomass (g)**	28.221	**<0.001**	36.428	**<0.001**	4.025	0.055
		**R/S ratio (g. g**^**-1**^**)**	0.804	0.378	3.261	0.082	0.360	0.553
		**RGR (g. g**^**-1**^**.day**^**-1**^**)**	26.529	**<0.001**	31.493	**<0.001**	0.004	0.952

**Fig 1:**
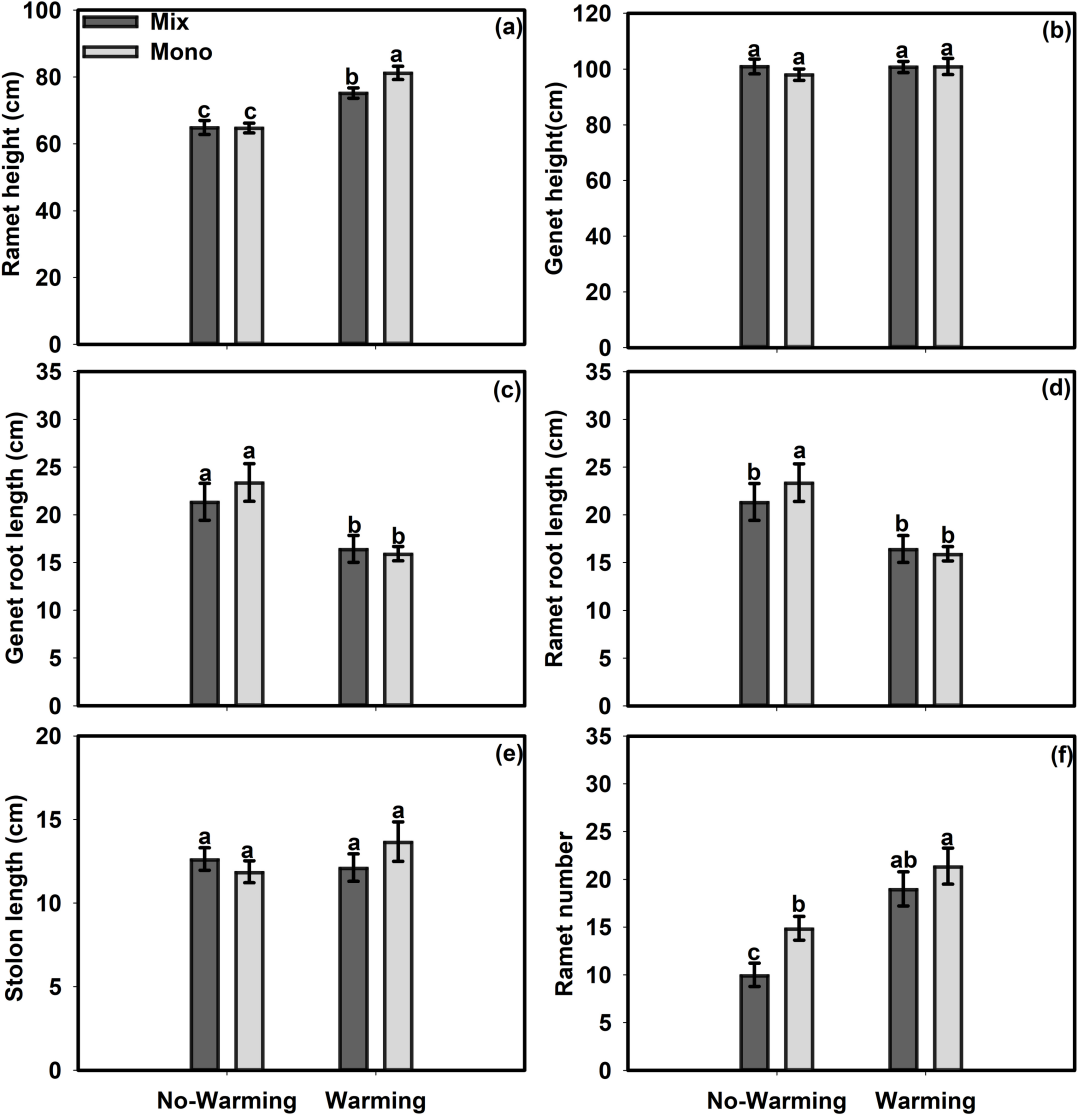
Effects of temperature and competition treatments on measures of morphology of *S*. *angustifolium*. (a) Ramet height, (b) Genet height, (c) Genet root length, (d) Ramet root length, (e) Stolon length, (f) Ramet number. Values are mean ± SE. Means with different small letters are significantly different at *P*<0.05 in different treatments.

**Fig 2:**
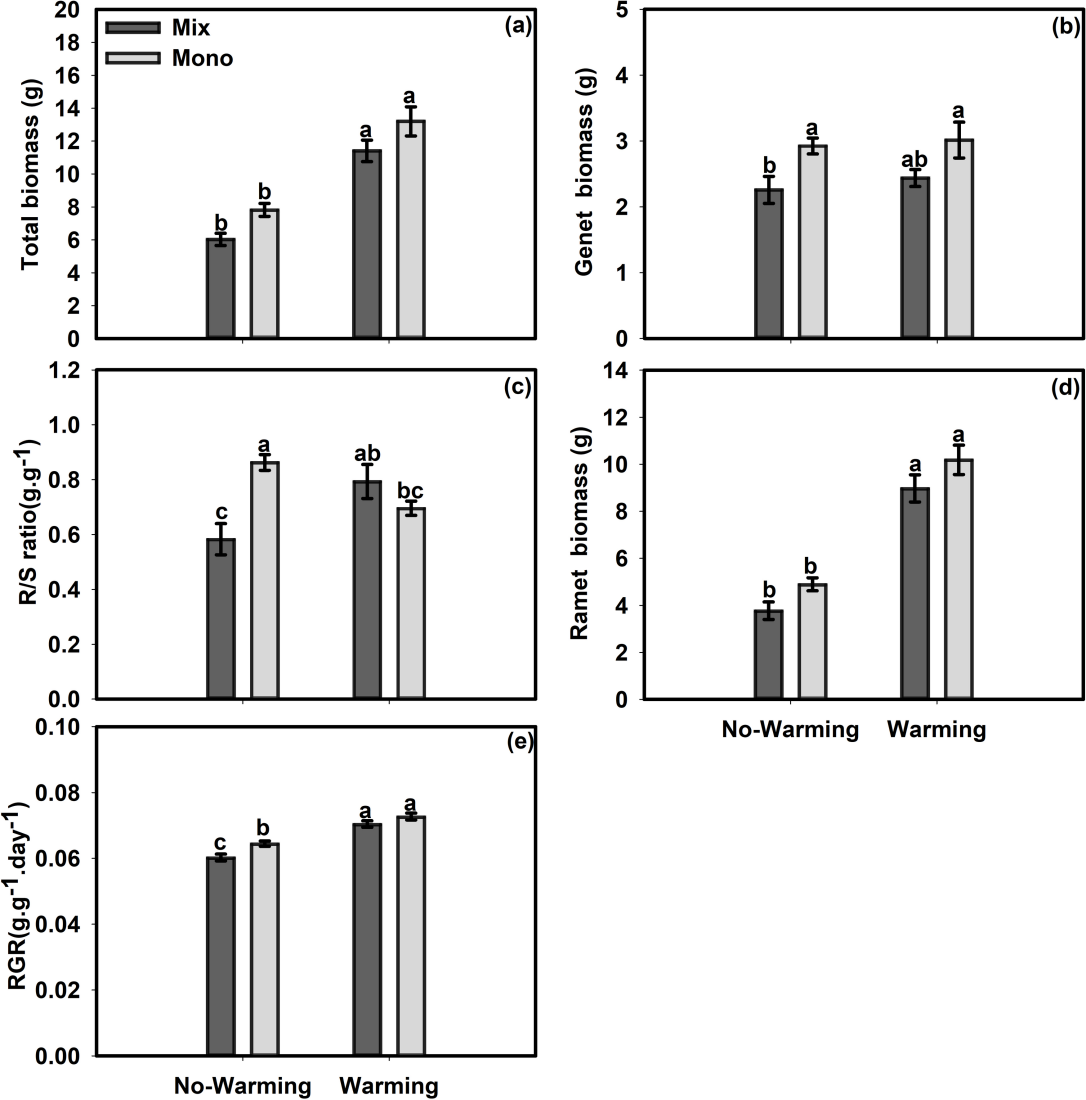
Effects of temperature and competition treatments on measures of biomass, the R/S ratio and the RGR of *S*. *angustifolium*. (a) Total biomass, (b) Genet biomass, (c) R/S ratio, (d) Ramet biomass, (e) RGR. Values are mean ± SE. Means with different small letters are significantly different at *P*<0.05 in different treatments.

Warming had a positive effect on the growth performance of ramets of *S*. *angustifolium*. Warming significantly increased the ramet height by 20.64%, the ramet number by 62.3%, the total biomass by 77.63%, the ramet biomass by 121% and the RGR by 14.77% (Figs 1A, 1F, 2A, 2D and 2E) but had no significant effects on the genet height, stolon length or genet biomass of *S*. *angustifolium* (Figs 1B, 1E and 2B). On the other hand, warming significantly decreased the genet root length by 27.68% and the ramet root length by 21.72% in *S*. *angustifolium* in the monoculture treatment (Fig 1C and 1D). Warming significantly increased the R/S ratio of the plants in the mixed culture treatment by 36.09% but decreased it by 23.97% for *S*. *angustifolium* in the monoculture treatment (Fig 2C). For *S*. *angustifolium*, the differences in ramet height in the no-warming control environment, in genet height, genet root length, ramet root length and ramet number in the warmer environment, and in stolon length between the mixed culture and monoculture treatments were not significant (Fig 1A–1F). The monoculture treatment significantly increased the ramet height by 8% in the warmer environment (Fig 1A). In the no-warming environment, the monoculture treatment increased the ramet root length of *S*. *angustifolium* by 79.91%, the ramet number by 48.75%, the genet biomass by 29.57%, the R/S ratio by 47.98% and the RGR by 6.99% (Figs 1D, 1F, 2B, 2C and 2E). The monoculture treatment had no significant effects on the total biomass or ramet biomass (Fig 2A and 2D).

**Fig 3:**
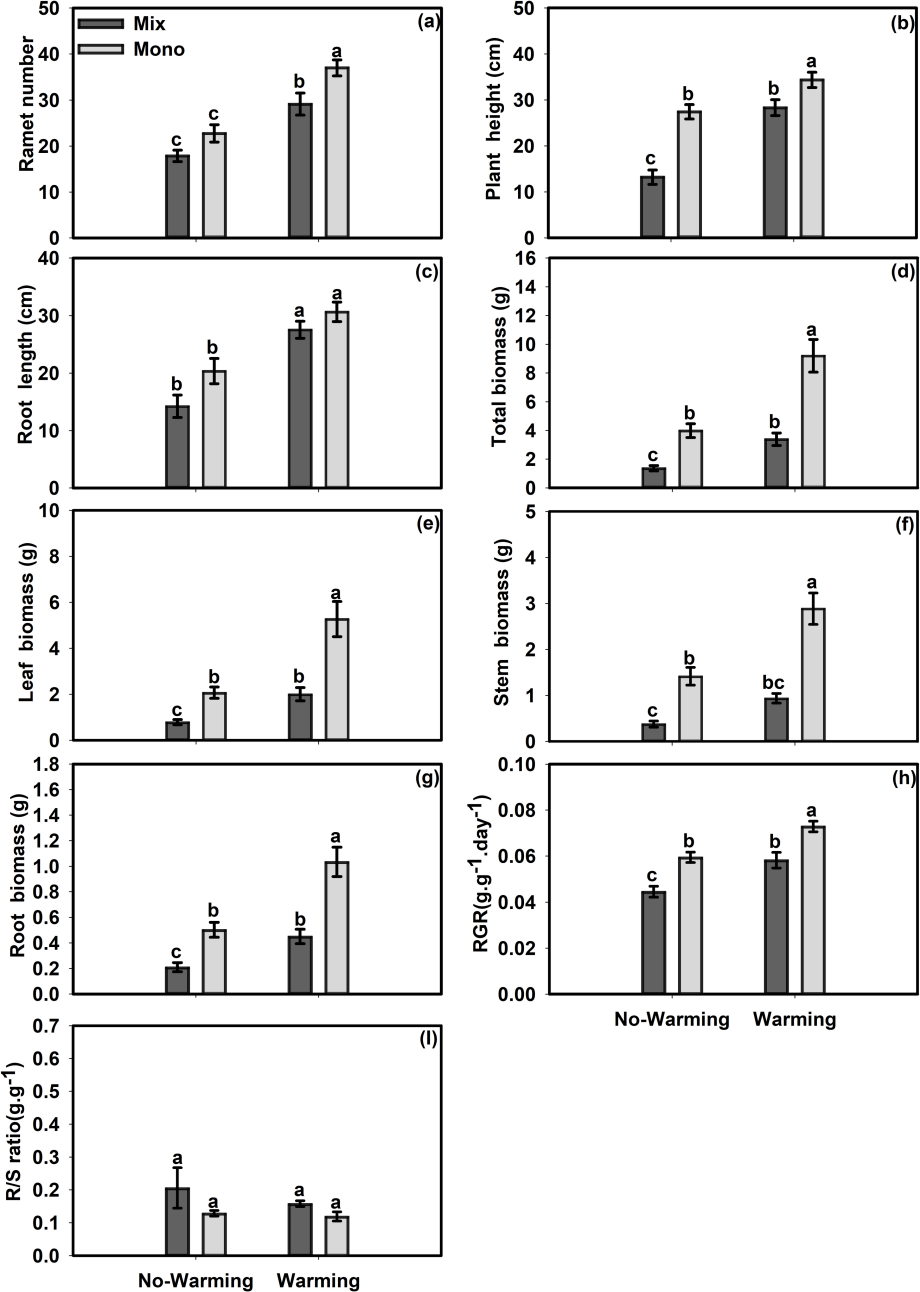
Effects of temperature and competition treatments on measures of biomass and morphology of *E*. *densa*. (a) Ramet number, (b) Plant height, (c) Root length, (d) Total biomass, (e) Leaf biomass, (f) Stem biomass, (g) Root biomass (h) RGR, (i) R/S ratio. Values are mean ± SE. Means with different small letters are significantly different at *P*<0.05 in different treatments.

Both temperature and competition had significant effects on all morphological and growth traits of *E*. *densa* except for the R/S ratio (Table 2). The ramet number, plant height, root length, total biomass, leaf biomass, stem biomass, root biomass and RGR of *E*. *densa *were 62.77%, 54.25%, 68.27%, 135%, 155%, 113%, 108% and 26.13% higher in the warming treatment than in the no-warming treatment, respectively (Fig 3A–3I). Competition significantly decreased the ramet number of *E*. *densa* by 27.13%, the plant height by 32.76%, the root length by 18.11%, the total biomass by 63.88%, the leaf biomass by 62.12%, the stem biomass by 69.3%, the root biomass by 57.4% and the RGR by 22.37% (Fig 3A–3I).

### The effect of the warming treatment on the intensity of competition

**Fig 4:**
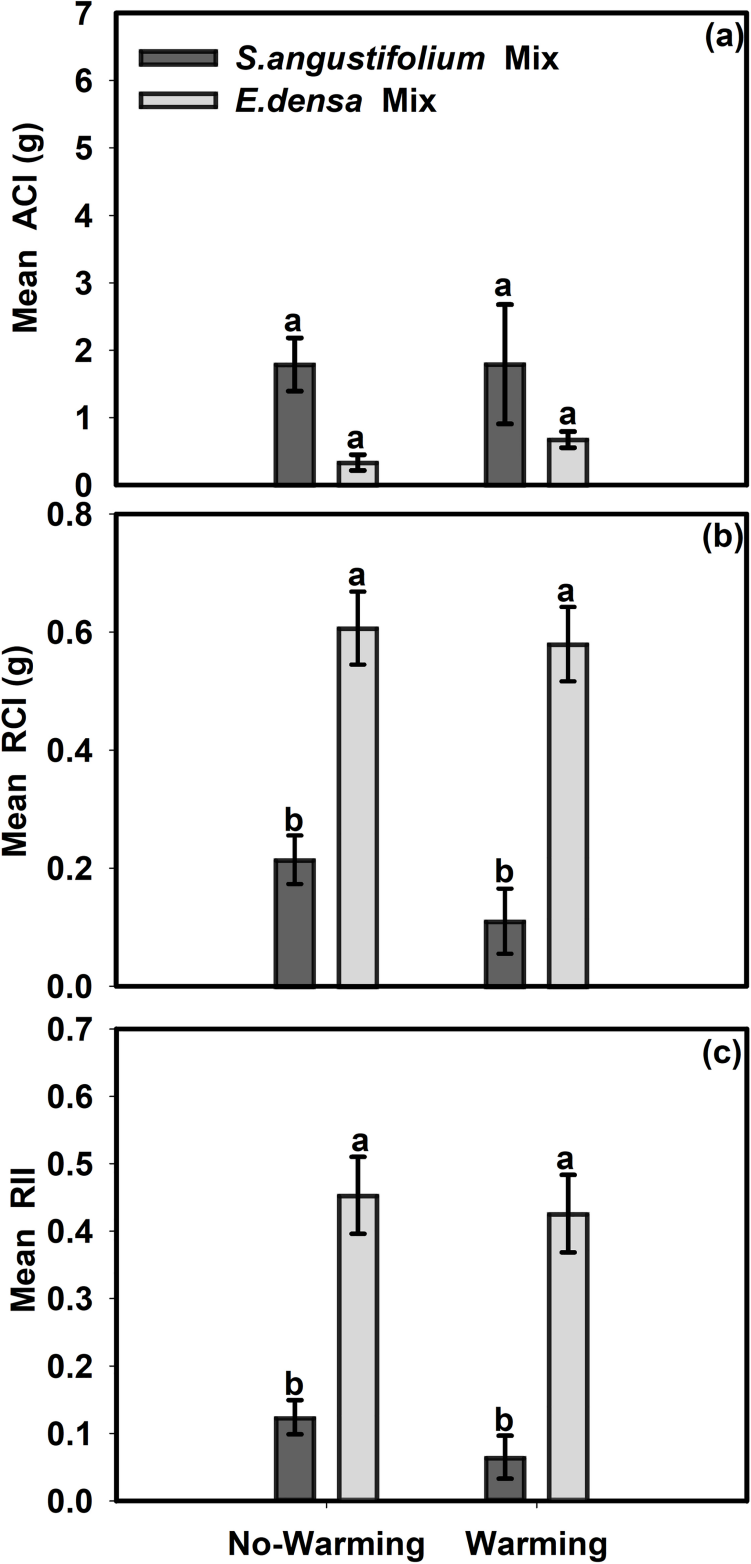
Mean absolute competition intensity, relative competition intensity and the relative interaction index for the treatment combination of temperature and competition for each plant species. (a) Mean ACI, (b) Mean RCI, (c) Mean RII. Values are mean ± SE. Means with different small letters are significantly different at *P*<0.05 in different treatments.

Species, but not temperature, had significant effects on the ACI, RCI and RII (Table 3). The warming treatment did not increase the interspecific competition between *E*. *densa* and *S*. *angustifolium*. In addition, the interaction between temperature and species showed no effect on the ACI, RCI or RII (Table 3). Unlike the ACI, the RCI and RII of *E*. *densa* in both the no-warming and warming treatments were two and three times greater than those of *S*. *angustifolium* (Fig 4A–4C). Thus, *E*. *densa* was more affected by interspecific competition.

**Table 3:** Two-way ANOVA results for effects of temperature and species on measures of ACI (Absolute competition intensity), RCI (Relative competition intensity) and RII (Relative interaction index).

	Species (S)		Temperature (T)		S x T	
	*F*_*1*,*28*_	*P*	*F*_*1*,*28*_	*P*	*F*_*1*,*28*_	*P*
**ACI**	5.078	**0.033**	2.956	0.097	0.523	0.476
**RCI**	50.698	**<0.001**	0.404	0.530	0.024	0.878
**RII**	47.683	**<0.001**	0.323	0.575	<0.001	0.996

## Discussion

High temperatures influence the flowering phenology, reproductive success, individual growth, and population dynamics of plants[47]. Previous studies have shown that the growth rates of *E*. *canadensis* and *Ranunculus aquatilis* strongly increase with temperature [48]. Our study indicates that high temperatures are also beneficial for some morphological and biomass traits and the RGR of *S*. *angustifolium*. In addition, *E*. *densa* was much more sensitive to increasing temperature[40]. The warmer temperature treatment significantly increased the functional traits of *E*. *densa* and promoted most growth traits, especially those pertaining to higher resource acquisition, for example, root length and plant height. These results suggest that the high temperatures caused by climate warming in the future will increase ramet formation and total biomass production in *S*. *angustifolium* as well as shoot branching and elongation and biomass production in *E*. *densa*.

Competition can significantly affect the performance of invasive plants and the success of invasion[49]. For example, competition eliminates the invasive advantage of *Eschscholzia californica *against native conspecifics[50]. In the current experiment, the mixed culture treatment had limited effects on the morphological and growth traits of *S*. *angustifolium*, while it significantly decreased most of the morphological and growth traits of *E*. *densa*. For example, the mixed culture significantly decreased the ramet height, genet biomass and R/S ratio in the warmer environment and decreased the ramet root length and number in the no-warming environment (Figs 2 and 3). These results suggest that the interspecies competition strongly influenced *E*. *densa*, whereas this competitive effect was less significant for *S*. *angustifolium*. The assessment of plant competition between species could help us understand how species coexist in the field and how it impacts the growth of plant species.

Experimental warming changes the hierarchies of competitive response and competitive effect, potentially leading to differential changes in growth, biomass production and coverage[29]. For example, experimental warming strongly weakened the competitive ability of *Pennisetum centrasiaticum *because of a reduction in the competitive response (i.e., the ability of a species to avoid being affected) hierarchy[29, 51]. Such changes in the competitive response hierarchy could potentially lead to changes in individual growth and community structure. In *S*. *angustifolium*, the warming treatment facilitated the investment of more resources into ramet biomass, particularly the aboveground portion of the ramet that developed slender floating leaves on the water’s surface. However, *E*. *densa* produced more ramets and formed dense canopies near the bottom of the water column in the elevated temperature treatment. Competition with *E*. *densa* had little effect on *S*. *angustifolium*, possibly because *S*. *angustifolium* shows different morphological characteristics for occupying space and exploring resources in different habitats. For example, *S*. *angustifolium* can establish roots in the sediment but develops long, linear leaves that float on the water’s surface or can extend above the water’s surface in wetland environments and shallow water[12, 33].

In conclusion, *S*. *angustifolium* occupies space and explores resources mainly through its ramets, and increased temperatures significantly promoted the growth traits of ramets; hence, the warming treatment significantly enhanced the competitive ability of *S*. *angustifolium*. Although the warming treatment significantly increased the performance of *E*. *densa*, interspecific competition significantly limited its growth traits. The main limiting factors for *E*. *densa* are high altitudes and cold-water springs[36]. Therefore, the warming climate in the future will further accelerate the promotion of *E*. *densa* invasion at high altitudes in northern China, although native species have a competitive advantage.

## Supporting information

S1 File Data files used in this study. This files contains all data used for this manuscript.
(XLSX)
